# Supplementary material for: Perinatal Distress and Depression in Culturally and Linguistically Diverse (CALD) Australian Women: The Role of Psychosocial and Obstetric Factors
Source: Int J Environ Res Public Health. 2019 Aug 16;16(16):2945. doi: 10.3390/ijerph16162945 (PMC6720521; doi:10.3390/ijerph16162945)
Supplement: Supplementary file 1 [file ijerph-16-02945-s001.pdf]

Table S1: Characteristics of the study population (N=25,407)

Study factors	n	(%)
**Psychosocial factors**		
**Maternal age group** (N=25,407)		
20-34 years	23,812	93.7
< 20 years	124	0.5
≥35 years	1,471	5.8
**SES category (N=23,786)**		
High	1,861	7.8
Middle	9,377	39.4
Low	12,548	52.8
**Supportive partner (N=21,672)**		
Yes	21,061	97.2
No	611	2.8
**Maternal history of childhood abuse (N=21,276)**		
No	20,525	96.5
Yes	751	3.5
**Pregnancy known to FACS (N=18,584)**		
No	18,444	99.25
Yes	140	0.75
**Previous child in OOHC (N=14,495)**		
No	14,009	96.6
Yes	486	3.4
**Antenatal depressive symptoms (N=20,560)**		
EPDS < 9	16,972	82.6
EPDS 10-12	2,078	10.1
EPDS >13	1,510	7.3
**Postnatal depressive symptoms (N=19,342)**		
EPDS < 9	17,425	90.1
EPDS 10-12	1,194	6.2
EPDS >13	723	3.7
**Psychosocial IPV (N=21,583)**		
No	21,238	98.4
Yes	345	1.6
**Physical IPV (N=21,523)**		
No	21,240	98.7
Yes	283	1.3
**Major nationality group (N=25,407)**		
Oceania	1,481	5.8
North-West Europe	384	1.5
Southern-Eastern Europe	1,316	5.2
North Africa & The Middle East	5,846	23.0
South-East Asia	6,222	24.5
North-East Asia	3,512	13.8
Southern & Central Asia	5,127	20.2
Americas	634	2.5
Sub-Saharan Africa	885	3.5
**Obstetrics factors**		
**Antenatal health problems (N=24,603)**		
No	20,517	83.4
Yes	4,086	16.6
**Alcohol use in pregnancy (N=22,373)**		
No	22,194	99.2

Yes	179	0.8
**Type of delivery (25,378)**		
Normal vaginal	15,004	59.1
Assisted vaginal	2,924	11.5
Caesarean section	7,450	29.4

SES: Socioeconomic status; OOHC: out of home care; FACS: Family and Community Services; IPV: intimate partner violence; EPDS: Edinburgh Postnatal Depression Scale
